# RESPONSIVENESS OF THE K-FORCE GRIP DYNAMOMETER IN PATIENTS WITH ICU-ACQUIRED WEAKNESS: A PROSPECTIVE LONGITUDINAL STUDY

**DOI:** 10.2340/jrm.v58.46235

**Published:** 2026-09-28

**Authors:** Ingrid D. VAN IPEREN, Marleen FLIM, Peter E. SPRONK

**Affiliations:** 1 Department of Intensive Care Medicine, Gelre Hospitals, Apeldoorn; 2 Expertise Center for Intensive Care Rehabilitation – ExpIRA, Apeldoorn, The Netherlands

**Keywords:** ICU-acquired weakness, hand strength, muscle strength dynamometer, critical illness, intensive care unit, rehabilitation, muscle weakness

## Abstract

**Objective:**

Handgrip strength is commonly used to assess muscle strength in critically ill patients, including those with intensive care unit-acquired weakness. While the reliability and validity of the K-force grip dynamometer have been established, its responsiveness remains unknown. This study evaluated the responsiveness of handgrip strength measured with the K-force grip dynamometer in intensive care unit-acquired weakness patients.

**Methods:**

In this prospective longitudinal study, intensive care unit-acquired weakness patients were assessed at baseline and after 1 week. Responsiveness was examined using effect sizes and standardized response means. The Medical Research Council sum score was included as a clinical comparator of global measure strength. Changes over time were analysed using Wilcoxon signed-rank tests.

**Results:**

Thirty-six patients were included. Dominant handgrip strength increased from 2650 g to 3450 g, with an effect size of *r* = 0.86 and standardized response means of 0.77 (*p* < 0.001). Non-dominant handgrip strength increased from 2,700 g to 3,783 g, with an effect size of *r* = 0.90 and SRM of 0.70 (*p* < 0.001). Medical Research Council sum score increased from 26.5 to 30.0 (*p* < 0.001).

**Conclusion:**

The K-force grip dynamometer demonstrated strong responsiveness in patients with intensive care unit-acquired weakness, supporting its use for detecting short-term changes in handgrip strength in critically ill patients.

## LAY ABSTRACT

Patients who spend a long time in the intensive care unit (ICU) often experience severe muscle weakness. Monitoring recovery of muscle strength is important to evaluate rehabilitation and guide treatment. Handgrip strength is a simple way to measure muscle strength, but it is important to know whether a measuring device can detect changes in strength as patients recover. This study examined the K-force grip dynamometer, a handheld device that measures handgrip strength. Thirty-six patients with muscle weakness after critical illness were assessed and reassessed 1 week later. The results showed that the device was able to detect improvements in handgrip strength over this short period. Patients demonstrated clear increases in grip strength, indicating that the device is sensitive to changes during recovery. These findings suggest that the K-force grip dynamometer may be a useful bedside tool for monitoring muscle strength in critically ill patients. This could help healthcare professionals track recovery and evaluate the effects of rehabilitation and other treatments aimed at improving muscle strength.

Muscle weakness is a common consequence of critical illness and frequently manifests as intensive care unit-acquired weakness (ICU-AW) (1). Patients with ICU-AW often experience delayed mobilization, prolonged mechanical ventilation, extended hospital stay, and reduced functional recovery (2). Because the time path of strength recovery can be slow and variable, clinicians could benefit from tools that can objectively detect meaningful changes in muscle strength over time (3).

Handgrip strength (HGS) is widely used as a proxy for overall muscle strength and functional status in the ICU (4). The test is quick to administer, non-invasive, and feasible at the bedside, even in patients with limited endurance or mobility (5). Serial measurements of grip strength may offer valuable insight into recovery trajectories and the effects of rehabilitation strategies (4).

In clinical practice, the Jamar dynamometer is most often used to quantify HGS and is commonly regarded as the reference standard (6–8). However, the device presents practical limitations in patients with severe weakness (9, 10). A relatively high force output is required to generate reliable measurements, which exceeds the capabilities of patients with ICU-AW (9). In addition, the Jamar lacks sufficient sensitivity to detect small but clinically relevant changes in strength, restricting its usefulness for longitudinal monitoring in this specific population.

The K-force grip dynamometer (K-invent, Montpellier, France) was developed as a lightweight and highly sensitive alternative, capable of detecting low force levels and subtle changes in grip strength. In a previous study, its feasibility, reliability, and validity were demonstrated in patients with ICU-AW, supporting its potential clinical applicability (5). To track progress in the rehabilitation trajectory of patients, an instrument should be able to detect genuine change over time (11). This aspect is particularly important in the ICU setting, where early improvements in muscle strength may be modest yet clinically meaningful. If a measurement tool is insufficiently responsive, relevant changes in patient status may go unnoticed, limiting its value for clinical decision-making (12).

To date, the responsiveness of the K-force grip dynamometer in patients with ICU-acquired weakness has not yet been established.

The aim of this study was to investigate the responsiveness of the K-force grip dynamometer in patients with ICU-acquired weakness, to determine whether the device is suitable for monitoring recovery and evaluating the effects of strategies aimed on improving muscle strength.

## METHODS

### Study population

Participants were recruited from the mixed medical and surgical ICU at Gelre Hospitals and the medical, surgical, and trauma ICU at Medisch Spectrum Twente. Eligible patients were at least 18 years of age, had been admitted to the ICU for more than 48h, and demonstrated muscle weakness, defined as a Medical Research Council sum score (MRC-SS) below 48, determined by a physiotherapist. In addition, the probability of ICU-AW was defined by the attending physician ruling out other causes for muscle weakness. Additionally, patients had to be awake, defined as a Richmond Agitation-Sedation Scale (RASS) score between –1 and +1 and oriented, defined as a negative Confusion Assessment Method for the ICU (CAM-ICU) score, ensuring that participants could reliably perform the assessments. Patients with neurological conditions affecting hand function were excluded.

This study was reviewed by the Medical Ethics Committee Isala Zwolle, the Netherlands (METC number 221007), which determined that the study was not subject to the Dutch Medical Research Involving Human Subjects Act (WMO). The study was conducted in accordance with the ethical principles of the Declaration of Helsinki and applicable Dutch privacy regulations, including the General Data Protection Regulation (GDPR). Written informed consent was obtained from all patients or their legal representatives prior to inclusion in the study.

### Study design

In this prospective longitudinal study, handgrip strength (HGS) using the K-force grip dynamometer and MRC-SS were measured at 2 time points, 1 week apart. During this interval, patients continued their usual physical therapy as per local protocols, which are based on the Dutch consensus statement for physiotherapy in the ICU (13). Physical therapy consisted of individualized mobilization exercises, including passive and active assisted range of motion activities, bedside functional training, in-bed cycling, and early mobilization.

A 1-week follow-up period was considered sufficient to allow clinically meaningful changes in HGS to occur in a substantial proportion of patients during this early recovery phase. And although a longer interval was judged to potentially increase the likelihood of observing change, this was considered less feasible due to potential discharge or transfer from the ICU.

### K-force grip dynamometer

HGS was measured using the K-force grip dynamometer (K-invent, Montpellier, France); a small and lightweight device designed for both assessment and rehabilitation of grip strength. The instrument is particularly sensitive at low force ranges, making it suitable for patients with severe ICU-AW. To optimize performance in this population, the devices used in this study were configured with customized low-force sensitivity settings. These configurations included amplification of the lower force range, reduction of sensor hysteresis and signal noise, and firmware adaptations to improve measurement precision at very low grip forces. These settings can be implemented in devices intended for comparable clinical applications. The device connects via Bluetooth to a tablet, providing real-time feedback through a dedicated app that displays graphical representations of performance. The K-force measures 40 × 45 × 120 mm, weighs 150 g, is CE certified in Europe, and FDA registered in the United States. Data were recorded in kilograms and converted to grams for analysis, with software version 2.23.0 operated on an Apple iPad (Apple Inc, Cupertino, CA, USA).

### Responsiveness

Responsiveness was evaluated according to the COSMIN framework and defined as the ability of an instrument to detect change over time in the construct to be measured (11, 14).

As no suitable gold standard, such as the Jamar dynamometer, is available for patients with severe ICU-acquired weakness, construct-based responsiveness was evaluated over a 1-week period in patients with ICU-AW. The 1-week period was chosen in resemblance to the Physical Function Intensive Care Test study of Nordon-Craft et al. (15), simultaneously aligning with the widely used clinical practice to set and evaluate rehabilitation interventions weekly. The MRC-SS was included as a complementary measure of global measure strength to provide clinical context for HGS changes. The MRC-SS is commonly used in critically ill patients and has demonstrated adequate interobserver reliability and sensitivity to detect muscle weakness, and evaluates 6 bilateral muscle groups with scores ranging from 0 (no contraction) to 5 (normal strength), resulting in a total score between 0 and 60 (2, 16–18).

HGS primarily reflects distal muscle function and is highly sensitive to short-term changes; the MRC-SS provides a broader clinical assessment of overall muscle strength across multiple muscle groups. Using both measures allowed us to evaluate changes in hand function in the context of general muscle recovery in patients with ICU-acquired weakness (18, 19).

HGS was measured using the K-force grip dynamometer following a standardized assessment protocol tailored to ICU-AW patients described previously (Appendix S1) (5). Patients were positioned semi-upright in bed, with a headrest angle of 30 to 45 degrees. The forearm was supported by the bed and the wrist was placed in a neutral position. Each hand was tested in 3 consecutive repetitions of 3 s each and 60 s of rest between repetitions. Participants were prompted verbally to squeeze the instrument as hard as possible until the researcher told them to stop. For each repetition, the maximum grip force was recorded, and the mean of the 3 maximum values was used for analysis. HGS and MRC-SS were assessed within the same clinical assessment period and as close in time as practically feasible at both baseline and follow-up.

In line with the construct approach, we hypothesized that HGS would show a statistically significant improvement over the 1-week period, reflecting at least moderate responsiveness with a Standardized Response Mean (SRM) ≥0.5. In addition, modest improvements in MRC sum scores were expected during the follow-up period, reflecting recovery of overall muscle strength.

### Statistical analysis

Paired samples *t*-tests or the Wilcoxon signed rank tests were used to compare baseline and follow-up measurements, depending on the normality of the change scores. Normality was assessed using the Shapiro–Wilk test. For normally distributed data, effect size (ES) was expressed as Cohen’s *d*, whereas for non-normally distributed data, rank biserial correlation (*r*) was reported. The standardized response mean (SRM) was calculated as the mean change divided by the standard deviation of the change scores, reflecting responsiveness relative to variability. Exploratory analyses of the relationship between changes in HGS and changes in MRC-SS were performed using Pearson or Spearman correlation coefficients, supported by scatterplots. All statistical analyses were performed using R (version 4.4.2; R Foundation for Statistical Computing, Vienna, Austria), with a significance level set at *p* < 0.05.

## RESULTS

A total of 36 patients meeting the inclusion criteria were enrolled in the study, of whom 32 were admitted to Gelre Hospitals and 4 to Medisch Spectrum Twente. Patient characteristics are presented in Table I. The study population consisted predominantly of male patients (63.9%) with a median age of 70 years (IQR 60.8–74.2). Most patients were admitted for medical conditions (66.7%), while 22.2% underwent emergency surgery and 11.1% elective surgery. At inclusion, patients demonstrated substantial muscle weakness, with a mean MRC sum score of 25 (13.0).The median ICU length of stay was 35 days (IQR 24.0–56.0), and the mean duration of mechanical ventilation was 31.0 days (SD 17.9). Follow-up measurements were unavailable for 3 patients due to death or transfer to another hospital.

**Table I:** Patient characteristics (*n* = 36)

Gender, *n*, %	
Male	23 (63.9)
Female	13 (36.1)
Age (median [IQR])	70.0 [60.8, 74.2]
Dominant hand, *n* (%)	
Left	3 (8.3)
Right	33 (91.7)
Total days of mechanical ventilation, mean (SD)	31.0 (17.9)
MRC-SS at inclusion, mean (SD)	25 (13.0)
Diagnosis at time of admission, *n* (%)	
Medical	24 (66.7)
Emergency surgery	8 (22.2)
Elective surgery	4 (11.1)
Length of ICU stay, median [IQR]	35.0 [24.0, 56.0]
ICU mortality, *n* (%)	
No	33 (91.7)
Yes	3 (8.3)
APACHE II (mean (SD)	21.9 (9.4)

MRC-SS: Medical Research Council sum score.

### Handgrip strength responsiveness

Responsiveness was assessed in 33 patients. In 2 patients, handgrip strength data were available for only 1 hand due to a painful and swollen left arm in 1 patient and insufficient attention to follow directions in the other patient. The median time between baseline and follow-up measurements was 7 days (IQR 7–7). Changes in handgrip strength and MRC sum scores over the 1-week follow-up period are presented in Table II. Significant improvements were observed in both dominant and non-dominant handgrip strength with large effect sizes, using rank biserial correlation, and SRM values indicating good internal responsiveness of the K-force grip dynamometer. Although improvements in MRC-SS were more modest, a statistically significant increase in overall muscle strength was also observed over time.

**Table II:** Responsiveness outcomes for handgrip strength and MRC sum scores after 1 week

Outcome	Baseline (*n* = 36) Median (IQR)	Follow-up (*n* = 33) Median (IQR)	Δ score Median (IQR)	*p*-value*	Effect size (*r*)	SRM
Dominant HGS (g)	2650 (767-5025)	3450 (1783–8008)	1117 (442-2633)	*p* < 0.001	0.86	0.77
Non-dominant HGS (g)	2700 (933–4733)	3783 (1767–6800)	1067 (417–2333)	*p* < 0.001	0.90	0.7
MRC-SS	26.5 (16.0-36.2)	30.0 (23.7–41.5)	4 (0.0–13)	*p* < 0.001		

HGS: Hand grip strength, MRS-SS: Medical Research Counsil sum score, SRM: Standardized Response Mean; IQR: interquartile range).

* *p*-values are calculated using the Wilcoxon signed-rank test.

External responsiveness analyses demonstrated weak correlations between changes in handgrip strength and changes in MRC-SS over the 1-week follow-up period. For the dominant hand, the correlation between ΔHGS and ΔMRC was very weak and not statistically significant (Spearman’s rho = 0.06, *p* = 0.757). Similarly, changes in non-dominant handgrip strength showed a weak, non-significant correlation with ΔMRC (Spearman’s rho = 0.14, *p* = 0.466). The results of the relationship between changes in HGS and changes in MRC-SS were visualized by scatterplots (Fig. 1).

**Fig. 1:**
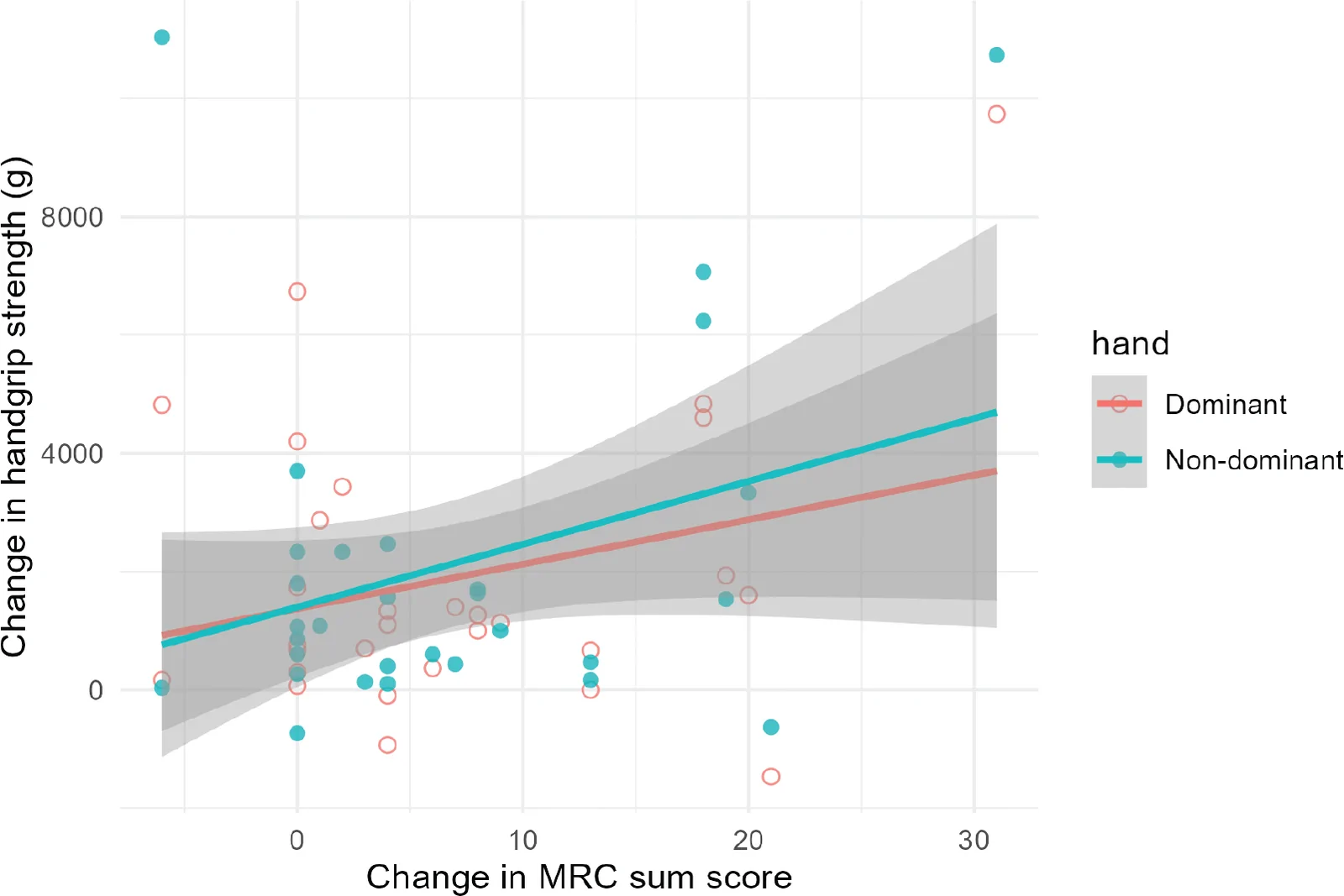
Scatterplot of ΔHGS vs ΔMRC sum score.

## DISCUSSION

The aim of this study was to evaluate the responsiveness of handgrip strength (HGS) measured with the K-force grip dynamometer in patients with ICU-acquired weakness (ICU-AW) (19). Establishing responsiveness is essential for determining whether HGS can be used to monitor recovery and guide rehabilitation in critically ill patients.

In this study, HGS demonstrated substantial improvements over a 1-week period in both dominant and non-dominant hands, supported by large effect sizes and standardized response means, indicating strong internal responsiveness of the K-force grip dynamometer. Conversely, changes in the Medical Research Council sum score (MRC-SS) were smaller but statistically significant. No meaningful correlation was observed between changes in HGS and MRC-SS.

Evidence specifically addressing the responsiveness of HGS in ICU-AW populations remains limited, particularly over short time intervals such as 1 week. Most previous studies have focused on cross-sectional associations between HGS and global muscle strength, or on its role in identifying ICU-AW, rather than its ability to detect longitudinal change (18–20).

In addition, concerning the minimal clinically important difference (MCID), no studies in critically ill patients could be identified. One systematic review selected 4 studies determining MCID for HGS, which ranged from 0.04 kg to 6.5 kg, but all used the Jamar dynamometer, and were performed in populations that provide no sensible comparators to our target group (21).

Similarly, we were not able to identify previous studies that evaluated the responsiveness of the K-force grip dynamometer in critically ill patients. As a result, direct comparison with existing literature on the same device or population was not possible.

Previous research has, however, demonstrated that HGS is a reliable and clinically relevant measure of muscle strength in critically ill populations (5, 22, 23). In addition, continuous strength measures are generally considered more sensitive to change than ordinal scales due to their greater measurement precision (14). The large changes and high responsiveness indications observed in the present study are therefore consistent with the expected behaviour of continuous force-based measures.

Previous studies have examined the relationship between HGS and the MRC-SS, mainly in terms of diagnostic agreement or identification of ICU-AW (3, 20, 22). However, these studies did not evaluate whether changes in HGS corresponded to changes in global muscle strength over time. To our knowledge, this is the first study to investigate longitudinal associations between ΔHGS and ΔMRC-SS in ICU patients.

Several factors may explain the strong responsiveness observed for HGS and the absence of a meaningful association with MRC-SS changes. First, HGS is a continuous measure and therefore more sensitive to small changes in muscle performance, whereas the MRC-SS is an ordinal scale with a limited number of response categories. This may reduce its sensitivity to detect subtle short-term changes (14).

Second, the restricted variability in MRC-SS change scores likely contributed to the weak association between the 2 measures. When change scores show limited dispersion, correlations are typically diminished even when underlying constructs are related (14).

Third, HGS and MRC-SS assess related but not identical constructs. HGS primarily reflects distal muscle strength, whereas the MRC-SS evaluates multiple proximal and distal muscle groups and therefore captures a broader but less sensitive construct of global muscle strength (3).

### Strengths and limitations

A key strength of this study is the hypothesis-driven evaluation of responsiveness in accordance with COSMIN methodology, ensuring a structured and transparent assessment of measurement properties. Both internal responsiveness and external responsiveness were assessed, providing a comprehensive evaluation of change over time. Another strength is the use of a standardized measurement protocol and a validated, highly sensitive dynamometer in a clinically relevant ICU-AW population, enhancing the reliability and applicability of the findings.

Finally, the short follow-up period allowed the assessment of early changes in muscle strength, which is particularly relevant after the acute phase of critical illness where identifying early recovery is clinically meaningful.

Several limitations should be acknowledged. First, the sample size was relatively small, allowing only exploratory conclusions regarding responsiveness, and possibly limiting generalizability. Second, we did only collect MRS-SS; in the light of evaluating upper limb function, the upper limb specific scores could have been used for more detailed analyses. Third, the 1-week follow-up period may have been insufficient to capture more substantial changes in global muscle strength, particularly as reflected by the MRC-SS.

Although a standardized protocol was used, muscle strength in critically ill patients may be influenced by multiple factors over short time periods, including true recovery from critical illness, but also daily fluctuations in activity and fatigue, which may not necessarily reflect sustained functional improvement.

### Recommendations for future research

Future research should include larger and more heterogeneous ICU populations to confirm the responsiveness of HGS across different clinical trajectories. Longer follow-up periods may help to better understand the relationship between changes in HGS and global muscle strength over time. In addition, future studies could compare HGS with alternative reference standards of muscle function, such as ultrasound-based muscle thickness measurements or electrophysiological assessments. These may provide more sensitive or mechanistic comparisons than ordinal clinical scales such as the MRC-SS.

In conclusion, handgrip strength measured with the K-force grip dynamometer demonstrated good responsiveness over a 1-week period in patients with ICU-acquired weakness. These findings suggest that handgrip strength may be a sensitive measure for detecting short-term changes in muscle strength in critically ill patients. However, further research is needed to confirm these findings in larger populations and to establish clinically meaningful thresholds for change.

## Supplementary Material


