# Supplementary material for: RESPONSIVENESS OF THE K-FORCE GRIP DYNAMOMETER IN PATIENTS WITH ICU-ACQUIRED WEAKNESS: A PROSPECTIVE LONGITUDINAL STUDY
Source: J Rehabil Med. 2026 Sep 28;58:46235. doi: 10.2340/jrm.v58.46235 (PMC13624958; doi:10.2340/jrm.v58.46235)
Supplement: Supplementary file 1 [file JRM-58-46235-s1.pdf]

Additional file 1: protocol handgrip measurements

Preparation

• Start with explaining the purpose of the measurements: "This test is done to measure your maximum hand grip strength."
• Ask the patient if they are left- or right-handed. If the dominant hand is known, start testing the dominant hand.
• The ideal position for the execution of the measurement is lying on the back with the headrest at an angle between 30 and 45 degrees (semi-upright position). If the patient is not in this position, ask the ICU nurse to assist in changing the position. If this is not possible, the most important instruction is that the patient should be lying on their back. Report patient and bedrest position.
• Next, demonstrate how to hold the instrument by stating: “I would like you to hold the instrument like this and then squeeze it as hard as possible.” The instrument is then given to the patient.
• Assist the patient in holding the instrument, ensuring that the top of the instrument is pointing upward and the arm is stretched out on the bed. It is important that the instrument rests on the bottom on the bed.
• Keep your hands close to the instrument to be able to catch it when the patient is unable to hold the instrument properly.
• If the patient is unable to hold the instrument sufficiently, the instrument may be supported by lightly placing two fingers on the top of the instrument.
• Then open the K-force app and select the patient.
• Ask the patient if they are ready to start the measurement and read through the following steps in preparation.

Measurement
• First, a test is performed where the patient is asked to squeeze the instrument for three seconds. If difficulties arise during this measurement, these can be resolved before the actual measurements begin.
• Ask the patient if they have any questions or comments before starting the measurement.
• Explain the steps of the measurement to the patient: “When I tell you to start squeezing, you should squeeze as hard as possible until I tell you to stop. I will ask you beforehand if you are ready and tell you when the measurement begins. Stop immediately if you experience any pain or discomfort at any time during the measurement.”
• The app is used for both the right and left hand. Make sure the dominant hand is measured first. The settings for the measurements are as follows:
Repetitions: 3
Duration (sec): 3
Rest (sec): 60
Preparation time: 10

• In the app, press "start measurement", where the patient has 10 seconds before they have to start squeezing.
• Instruct the patient to start squeezing by counting down "3, 2, 1, start!".
• During the three seconds of the measurement, encourage the patient by saying "harder, harder, harder".
• Inform the patient that they can stop squeezing when the three seconds are up.
• Repeat the same instruction for the second and third measurements, and then for the other hand.
• Allow 1 minute of rest between each measurement to allow the patient to recover from the effort.
• After the first measurement, ask the patient if everything is going well and if the patient feels any discomfort during the measurement. If the measurement causes too much discomfort for the patient, further measurements are stopped. Later in the day, the measurement is repeated to see if it goes better. If the measurement still fails after three attempts, no further attempts are made, and the patient is excluded from the study.
• Export the generated data and email your work email address.
• Save the data using the patient ID
• Report any deviations from the protocol, patient comments, and any other concerns
